# Establishment of adult right ventricle failure in ovine using a graded, animal‐specific pulmonary artery constriction model

**DOI:** 10.1002/ame2.12124

**Published:** 2020-06-14

**Authors:** Michael Nguyen‐Truong, Wenqiang Liu, June Boon, Brad Nelson, Jeremiah Easley, Eric Monnet, Zhijie Wang

**Affiliations:** ^1^ School of Biomedical Engineering Colorado State University Fort Collins CO USA; ^2^ Veterinary Teaching Hospital Colorado State University Fort Collins CO USA; ^3^ Department of Clinical Sciences Colorado State University Fort Collins CO USA; ^4^ Department of Mechanical Engineering Colorado State University Fort Collins CO USA

**Keywords:** animal models, fibrosis, pulmonary artery banding, pulmonary hypertension, right heart failure

## Abstract

**Background:**

Right ventricle failure (RVF) is associated with serious cardiac and pulmonary diseases that contribute significantly to the morbidity and mortality of patients. Currently, the mechanisms of RVF are not fully understood and it is partly due to the lack of large animal models in adult RVF. In this study, we aim to establish a model of RVF in adult ovine and examine the structure and function relations in the RV.

**Methods:**

RV pressure overload was induced in adult male sheep by revised pulmonary artery constriction (PAC). Briefly, an adjustable hydraulic occluder was placed around the main pulmonary artery trunk. Then, repeated saline injection was performed at weeks 0, 1, and 4, where the amount of saline was determined in an animal‐specific manner. Healthy, age‐matched male sheep were used as additional controls. Echocardiography was performed bi‐weekly and on week 11 post‐PAC, hemodynamic and biological measurements were obtained.

**Results:**

This PAC methodology resulted in a marked increase in RV systolic pressure and decreases in stroke volume and tricuspid annular plane systolic excursion, indicating signs of RVF. Significant increases in RV chamber size, wall thickness, and Fulton's index were observed. Cardiomyocyte hypertrophy and collagen accumulation (particularly type III collagen) were evident, and these structural changes were correlated with RV dysfunction.

**Conclusion:**

In summary, the animal‐specific, repeated PAC provided a robust approach to induce adult RVF, and this ovine model will offer a useful tool to study the progression and treatment of adult RVF that is translatable to human diseases.

## 1 INTRODUCTION

Right ventricle failure (RVF) is associated with serious cardiac and pulmonary diseases that contribute significantly to the morbidity and mortality of patients.^1^ The prevalence of RVF is significantly increased in the later stages of pulmonary hypertension (PH), congenital heart disease (CHD), and left heart failure with preserved ejection fraction (HFpEF).^2^, ^3^, ^4^ Moreover, the mortality rate of these patients has not improved despite proposed therapeutic interventions.^3^, ^4^, ^5^, ^6^ The lack of effective treatment can be attributed to the incomplete understanding of the mechanisms of RVF and the lack of robust large animal models in adult RVF.^7^, ^8^, ^9^

Preclinical (animal) models are powerful tools to investigate various human diseases including RVF.^7^ Compared to small animal models, large animal models better mimic human physiology and pathophysiology^10^, ^11^, ^12^, ^13^ and thus are advantageous in studying both the pathogenesis and potential therapeutics that are more translatable to human patients.^14^ To date, various methods have been used to establish RV pressure overload, the most common etiology of RVF. These methodologies include pulmonary artery (PA) (Table 1) or pulmonary vein banding,^13^, ^15^ thromboembolic induction,^11^, ^16^, ^17^ chronic hypoxia,^18^ monocrotaline,^19^, ^20^ and the combination of sugen and chronic hypoxia.^21^, ^22^, ^23^ However, some of these models are essentially the models of PH, which mainly focus on the pulmonary vascular disease and do not necessarily involve the establishment of RVF. (Reviews of the PH models are cited here.^24^, ^25^, ^26^, ^27^)

**Table 1:** Review of prior small and large animal models of pulmonary arterial (PA) banding/constriction. All studies adopted the same degree of constriction (ie, with a fixed diameter, area reduction, or pressure level) except for Leeuwenburgh et al,^30^, ^31^, ^32^, ^34^ Ramos et al,^58^ Gold et al,^59^ Gaynor et al,^60^ and Verbelen et al,^35^ which used the same criteria as our study to elevate the RV pressure to the individual's systemic pressure

Study	Animal	Weight/age	Method	Application	Mortality Rate (%)	Model Duration
Heitmeier et al, 2019^61^	Mice	20‐25 kg/12 wk (adult)	Titanium clip around PA; reduce cross‐sectional area to about 66% of original area	Assess ubiquitin proteasome system in right heart hypertrophy; No RVF reported	N/A	3 wk
Kuroha et al, 1991^62^	Rat	2‐month, 7‐month, and 18‐month old (non‐adult and adult)	Silk thread around PA; increase of RV pressure by 15 mm Hg in each animal	Effect of age on RV hypertrophy due to RV pressure overload; No RVF reported	N/A	3 wk
Schou et al, 2007^63^	Rat	150‐200 g (adult)	Pulmonary trunk clip; compressed to outer diameter of 0.9 mm	Establish rat model of right‐sided heart failure and characterize systemic and cardiac changes	N/A	17 wk
Bogaard et al, 2009^64^	Rat	200 g (adult)	PA silk thread constriction; tightened to outer diameter 18G needle	Investigate if pressure overload alone can explain RVF associated with pulmonary hypertension; No RVF reported with PA constriction	N/A	6 wk
Hill et al, 2014^65^	Rat	8 wk (adult)	PA surgical clip; uniform RV pressure of 45‐50 mm Hg	Structural and mechanical adaptations of RV free wall; No RVF reported	N/A	3 wk
Hirata et al, 2015^42^	Rat	240‐260 g (adult)	PA clip or suture ligation; tightened to the outer diameter of an 18G needle	Comparison of methods to constrict PA; signs of RVF indicated by fibrosis and reduced TAPSE but not CO	22	8 wk
Jang et al, 2017^66^	Rat	8 wk (adult)	PA surgical clip; RV maximum systolic pressure > 50 mm Hg	RV biomechanical and hemodynamic changes under pressure overload; No RVF reported	N/A	3 wk
Wang et al, 2017^67^	Rat	Neonatal	PA constriction with nylon; tightened to outer diameter of 30G needle	Study of pathophysiological remodeling of RV due to congenital heart disease with RV afterload	25	7 d
Chery et al, 2019^68^	Rat	200‐225 g (adult)	PA suture over 18 G tube; PA band peak gradient of 25‐60 mm Hg	Human neonatal thymus stem cell therapy for RV; No RVF reported	33	100 d
Axelsen et al, 2019^69^	Rat	112 ± 12 kg (non‐adult)	Titanium clip; set to inner diameter of 0.7 mm	Assess treatment of pulmonary hypertension with 6‐mercaptopurine	5	7 wk
McKellar et al, 2015^70^	Rabbit	2.0‐2.5 kg (non‐adult)	Weekly PA banding with cuff; RV end systolic pressure > 25 mm Hg	To establish chronic, reversible RVF model to study RVF progression and recovery; RVF indicated by RV pressure and morphology, septum position, and histology only	Exact rate unknown; several out of 15 died	43 ± 1.6 d banding or 16.6 ± 3.3 d recovery post‐RVF
Ramos et al, 2018^58^	Rabbit	3.00 ± 0.23 kg (non‐adult)	Adjustable PA banding with C‐shaped ring; weekly inflations to achieve systemic pressures	Early and late cardiac remodeling due to RV pressure loading and therapy with endothelin‐1 receptor blockers; No RVF reported	N/A	3‐6 wk
Gold et al, 2019^59^	Rabbit	3.00 ± 0.23 kg (non‐adult)	Adjustable PA banding device (C‐shaped ring); weekly PAB inflations to achieve systemic RV pressures by day 21	Relationship between RV wall stress, fibrosis, and function under RV pressure loading; No RVF reported	N/A	3‐6 wk
Hsieh et al, 1992^29^	Dog	18‐23 kg (adult)	PA banding; increase of RVSP to 50 mm Hg at the end of first month and then by 20 mm Hg monthly increase, if necessary	RVF confirmed by fraction shortening decrease and RV dilation. Study the reversibility of right heart failure	14	3 mo PAB and additional 4 mo recovery
Gaynor et al, 2005^60^	Dog	20‐25 kg (adult)	PA banding; Weekly 0.3 to 0.5 mL of saline injection (ΔRVP ~ 10‐20 mm Hg) to achieve near‐systemic pressures	RA and RV hemodynamic adaptations to RV pressure overload; No RVF established	N/A	3 mo
Barbera et al, 2000^33^	Ovine	121 ± 1 day gestation	Inflation of vascular occluder around PA to increase RVSP by ~ 10‐30 mm Hg over first 3 d of pressure loading	Assessment of myocyte maturation due to pressure load in fetal ovine	N/A	10 d
Hon et al, 2001^71^	Ovine	3 mo (non‐adult)	PA ligation with band; PA systolic pressure > 60 mm Hg	Acute effects of overload on RV contractile function; No RVF reported	N/A	30 min
Leeuwenburgh et al, 2001^32^	Lamb	2‐3 wk (non‐adult)	PA constriction with an adjustable occluder for up to 12‐week period; RVSP matched to systolic pressure	Evaluation of biventricular systolic function; No RVF reported	23	64 ± 8 d
Leeuwenburgh et al, 2002^31^	Lamb	2‐3 wk (non‐adult)	PA constriction with an adjustable occluder for up to 12‐week period; RVSP matched to systolic pressure	Evaluation of biventricular diastolic function; No RVF reported	23	64 ± 8 d
Leeuwenburgh et al, 2003^30^	Lamb	2‐3 wk (non‐adult)	PA constriction with an adjustable occluder for up to 12‐week period; RVSP elevated to systemic level	Test feasibility of a device for PA constriction as a treatment in children with congenital heart disease; No signs of heart failure	23	64 ± 8 d
Leeuwenburgh et al, 2008^34^	Lamb	2‐3 wk (non‐adult)	PA constriction with an adjustable occluder up to 12‐week period; RVSP matched to systolic pressure	Evaluation of cellular and biochemical myocardial response; No RVF reported	23	64 ± 8 d
Yerebakan et al, 2009^72^	Ovine	4 mo (non‐adult)	3 mm Dacron band on pulmonary trunk; Elevation of RVSP to 50%‐60% above baseline	Acute and chronic response of RV to pressure and volume overload; No RVF reported	N/A	Immediately after PAB or 3 mo
Verbelen et al, 2015^35^	Ovine	10.5 ± 0.8 mo (adult)	PA constriction as much as was hemodynamically tolerated	Test ventricular assist device for pressure overloaded RV	N/A	10 min
Malinowski et al, 2018^10^	Ovine	50‐60 kg (adult)	PA occluder; increase RV peak pressure to > 150% of pre‐occlusion value	To establish acute RVF model with functional tricuspid regurgitation; No RVF with PA banding alone	N/A	15 min
Gufler et al, 2019^73^	Ovine	25 wk (adult)	PA banding; target maximal RVSP set to 50%‐60% above baseline	Adaptive response of RV to chronic pressure overload; No RVF reported	N/A	3 mo
Corno et al, 2003^74^	Porcine (mini‐pig)	18.2 ± 0.1 wk; 8.6 ± wk (non‐adult)	Adjustable PA band; perimeter range = 23‐30 mm	Evaluation of FloWatch (implantable device for PA banding) as a treatment for congenital heart disease; No RVF reported	N/A	24 wk; 10 wk

Compared to other RV pressure overload models, the PA banding/constriction (PAB or PAC) model is a model of RV adaptation or dysfunction alone with no pulmonary vascular diseases. While this model has been critiqued less realistic than the other PH models, it is unique and advantageous since the changes in the RV are the sole effect of the hemodynamic insult, that is, the pressure overload. Such a model provides us an opportunity to investigate the biomechanical mechanism of RV failure without other confounding factors such as altered systemic inflammation from pulmonary vascular diseases.^28^ PAB/PAC has been used in different animal species and with a mix of ages (from newborn to young adult) for RV adaptation or RVF studies (Table 1). To our knowledge, the only large animal study of chronic, adult RVF was performed in canine in the early 1990s, while the clinical standard of RVF was absent at the time.^29^ Moreover, both adult and non‐adult large animals have been used in the literature, with mixed goals of studying pediatric or adult RV diseases, as well as using PAC as a treatment option or means to induce RV dysfunction. For instance, lambs were commonly used and the response of the RV was associated with CHD.^30^, ^31^, ^32^, ^33^, ^34^ In adult ovine studies, it was the acute changes in the RV that were examined and the chronic remodeling and outcomes were not studied.^10^, ^35^ Therefore, despite the “apparently” widely used PAC model in large animals, to date, no chronic RVF has been established in adult ovine.

The goal of the present study is to adopt an animal‐specific, graded pressure overload method to establish chronic RVF in adult ovine and to investigate the structural and functional changes with RVF development. Ovine were chosen due to the widely reported similarities between human and ovine cardiovascular anatomy, function, and physiology.^14^, ^16^, ^25^ Our data suggest that the revised PAC method led to RVF development in sheep and can serve as a large animal model of chronic, adult RVF.

## 2 METHODS

### 2.1 Animal‐specific and graded pulmonary artery constriction (PAC) in ovine

All procedures were approved by Colorado State University Institutional Animal Care and Use Committee. Prior to surgery, 8‐month old male sheep (n = 3) were placed under general anesthesia with 3.3‐5 mg/kg ketamine and 0.1 mg/kg midazolam. During the surgical procedure, animals received 15 mcg/kg/min of ketamine and 35 mcg/kg/min of lidocaine. To induce RV pressure overload, an adjustable hydraulic occluder (AUS‐PORT 12x14 mm, Norfolk Vet Products, IL) was placed around the main pulmonary artery and secured with two 2‐0 polybutester sutures. Next, under pressure monitoring, saline was injected to the occluder acutely until the RV systolic pressure (RVSP) reached an equivalent number of its left ventricle (LV) systolic pressure as described previously.^30^, ^31^, ^32^, ^34^ The amount of saline was recorded and the saline was withdrawn from the occluder to allow the animal to recover from the surgical procedure for 2 days. This minimized the “surgical insult” to the RV and thus the response was mainly a result of hemodynamic overload induced via saline injections starting 2 days post‐surgery. Besides the baseline measurements in the PAC sheep, age‐matched, healthy intact sheep (n = 3) were used as additional controls (CTL).

**Figure 1:**
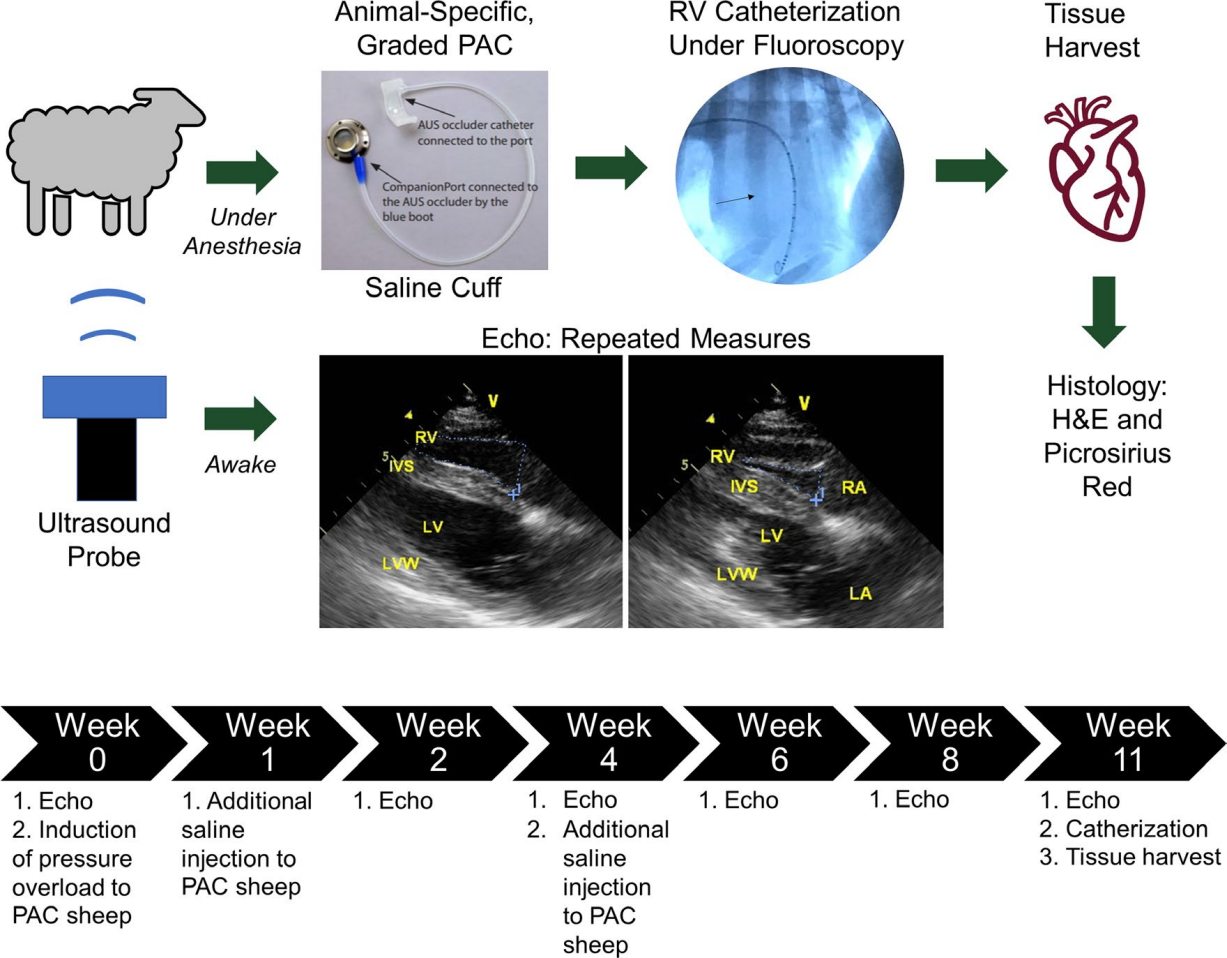
Visualized workflow of PAC sheep model and timeline of study. Saline cuff figure reproduced with permission from Norfolk Vet Prodcuts (https://norfolkvetproducts.com/wp‐content/uploads/2019/03/NVP_Catalog_2019‐01_email.pdf). Representative 4‐chamber view images obtained by echocardiography (echo) are from a PAC sheep at week 2 (left: diastole; right: systole)

Animal‐specific, graded filling of the occluder with saline was induced in awake animals at weeks 0, 1, and 4 post‐surgery (Figure 1). The amount of saline injections was determined by the procedure described above as well as the RV morphology and function from bi‐weekly echocardiography. If we observed signs of heart failure (eg, difficulty in breathing, anorexia, grinding teeth, etc), continuous RV dilation and RV hypertrophy (eg, increased RV area or RV wall thickness), or function decline (eg, decreased flow velocity across the PA valve and decreased TAPSE), we reduced the injection volume or did not inject any saline further (Table 2).

**Table 2:** Volumes of saline injections for the PAC animals at three time points over the 11‐week study

	Week 0	Week 1	Week 4	Total
Sheep 1 Injection (mL)	0.4	0.4	0.1	0.9
Sheep 2 Injection (mL)	0.6	0.6	0	1.2
Sheep 3 Injection (mL)	0.2	0.4	0.1	0.7

### 2.2 Echocardiography

Transthoracic two‐dimensional echocardiography was performed bi‐weekly (weeks 0, 2, 4, 6, 8, 11) using a 2.5 MHz transducer on a GE Vivid 7 (GE Healthcare) ultrasound machine. Briefly, parasternal images were obtained in the awake sheep in lateral recumbency, using American Society of Echocardiography guidelines with minor imaging plane modifications in the sheep.^36^ Ventricular dimensions (such as RV area, septum diameter, and RV or LV inner diameter; RVID/LVID), tricuspid annular plane systolic excursion (TAPSE), and flow dynamics were measured.

### 2.3 Hemodynamic measurements and terminal procedure

Prior to euthanasia, the CTL and PAC animals were anesthetized and RV catheterization was performed to obtain hemodynamic measurements. A 7 Fr Swan Ganz catheter (Edwards Lifesciences Corporation) was floated to the RV through the jugular vein and cranial vena cava under pressure monitoring and fluoroscopic guidance. Cardiac output (CO) and stroke volume (SV) were measured using the thermodilution method.^11^, ^13^ Finally, the RV systolic pressure was obtained by a pressure‐volume catheter (Millar, Houston, TX). Immediately following the hemodynamic measurements, the animals were euthanized with pentobarbital (IV) at 88 mg/kg and the hearts were harvested. RV tissue hypertrophy was measured by the wet weight, Fulton index (RV/(LV + Septum)), and wall thickness using a digital caliper.

### 2.4 Structural measurements

RV samples from the center of the anterior RV free wall were fixed in 10% formalin. Specimens were then dehydrated, embedded in paraffin, sectioned, and stained with H&E for cardiomyocyte morphology and Picro Sirius Red for collagen fibers. Cardiomyocyte morphology was imaged by an inverted microscope (Motic AE31E) and quantified with AmScope software (AmScope); collagen content and fiber orientation were imaged under polarized light by a transmission microscope (Nikon Eclipse E800) and quantified with Image Pro Premier software (Media Cybernetics). Color thresholding method was used to measure type I collagen, type III collagen, and non‐collagen areas, respectively.^18^, ^37^

### 2.5 Statistical and correlation analysis

One‐way ANOVA with repeated measures and Dunnett's post hoc tests were performed by Prism (GraphPad Software) to examine the functional changes in the RV during the PAC. Unpaired Student's *t* test was performed between the CTL and PAC groups in Excel (Microsoft). Pearson correlation analysis was used to investigate the correlations between the structural and functional properties. Data are presented as mean ± SD. *P* < .05 was considered statistically significant and 0.05 ≤ *P* < .1 was considered a trend.^38^, ^39^, ^40^ *r* > .8 was considered as a strong correlation.

## 3 RESULTS

### 3.1 Hemodynamic and functional changes in the RV with PAC

**Figure 2:**
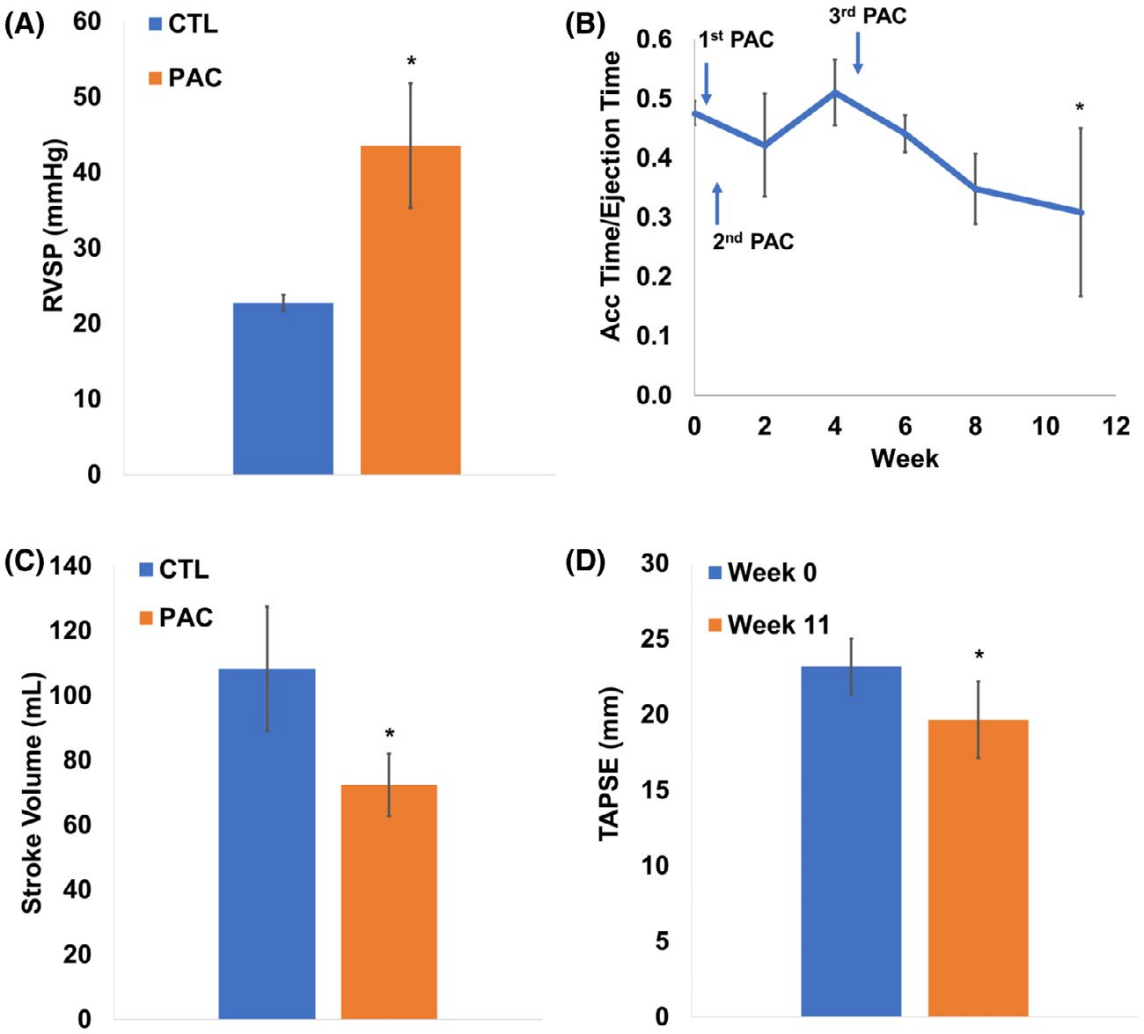
Decline in RV hemodynamics and function with PAC. A, Increase in RV systolic pressure. B, Decrease in pulmonary artery acceleration time/ejection time. C and D, Decrease in stroke volume and TAPSE in PAC sheep. *P < .05 vs. CTL group or Week 0

Eleven weeks post‐PAC, there was a significant increase in RVSP compared to the CTL group and a gradual decrease in the ratio of pulmonary artery acceleration time/ejection time (PA AT/ET) over time, with a significant reduction at the end point (Figure 2A,B, *P* < .05). These results indicate the successful induction of pressure overload and establishment of RVF in the sheep. Moreover, there was a significant decrease in SV in the PAC group compared to the CTL group at 11 weeks (Figure 2C, *P* < .05). Lastly, we found that TAPSE was significantly decreased at week 11 compared to the baseline (Figure 2D, *P* < .05). These results indicate that pressure elevation was successful, and RVF was evident in these animals.

### 3.2 Morphological changes in the RV with PAC

**Figure 3:**
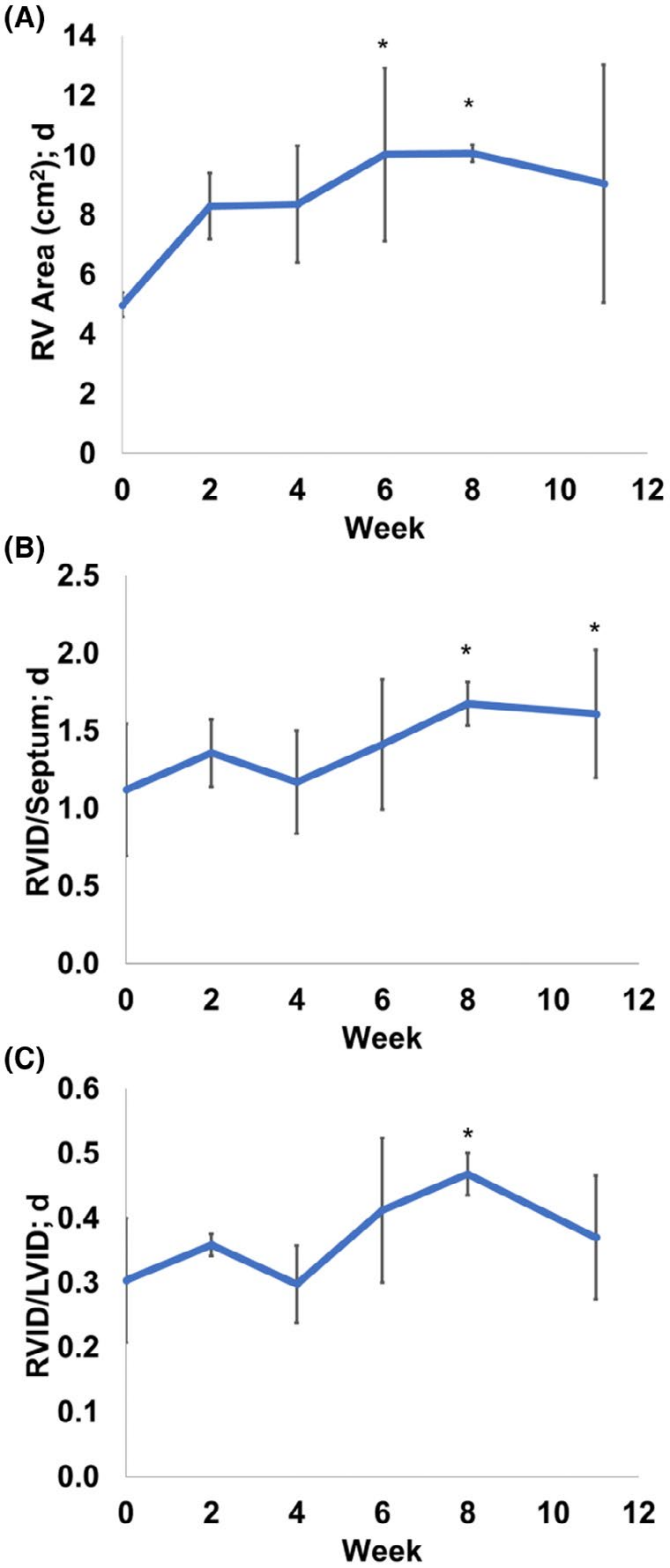
Temporal changes in diastolic geometry of the RVs over 11 wk of pressure overload (PAC). PAC led to gradual increases in (A) RV diastolic area, (B) RV inner diameter/septum diameter at diastole, and (C) RV inner diameter/LV inner diameter at diastole. *P < .05 vs. Week 0

With the chronic pressure overload, there were significant increases in diastolic RV area and RVID as measured by echocardiography (Figure 3), suggesting a progressive dilatation of the chamber. Some global changes in the hearts were examined after tissue harvest (Table 3). Both the RV weight/body weight (*P* < .05) and wall thickness (*P* < .05) were larger in the PAC group, and the Fulton index as a routine RV hypertrophy index was significantly increased as well (*P* < .05).

**Table 3:** Overall structural changes in ovine hearts with PAC. Data are presented as mean ± SD

	CTL	PAC
Body weight (kg)	81.6 ± 12.2	84.8 ± 4.5
RV weight (g)	61.7 ± 10.9	84.1 ± 13.1
RV weight/body weight (g/kg)	0.8 ± 0.0	1.0 ± 0.1*
Fulton index (%)	28.4 ± 1.3	43.0 ± 3.5*
RV wall thickness (mm)	5.9 ± 0.2	7.4 ± 0.6*
Myocyte width (μm)	13.5 ± 1.2	17.1 ± 0.7*
Collagen fiber angle (degrees)	54.0 ± 8.0	57.0 ± 12.0
Type I collagen content (%)	2.9 ± 1.0	4.3 ± 1.3
Type III collagen content (%)	0.7 ± 0.1	1.8 ± 0.7**
Total collagen content (%)	3.6 ± 1.0	6.1 ± 1.4

* *P* < .05; **P = .05.

### 3.3 Structural changes in the RV with PAC

From the H&E staining, we quantified RV cardiomyocyte width and found that there was a significant increase in the cell width with PAC (Table 3, *P* < .05). From the Picro Sirius Red staining, we examined RV collagen content and fiber orientation in the CTL and PAC groups using polarized microscope images. PAC tended to lead to more collagen accumulation (*P* = .065), especially type III collagen accumulation in the RV (Table 3, *P* = .05). There was no difference in collagen fiber orientation between the CTL and PAC groups (Table 3).

### 3.4 Correlation analyses of the structure and function in the RV

**Figure 4:**
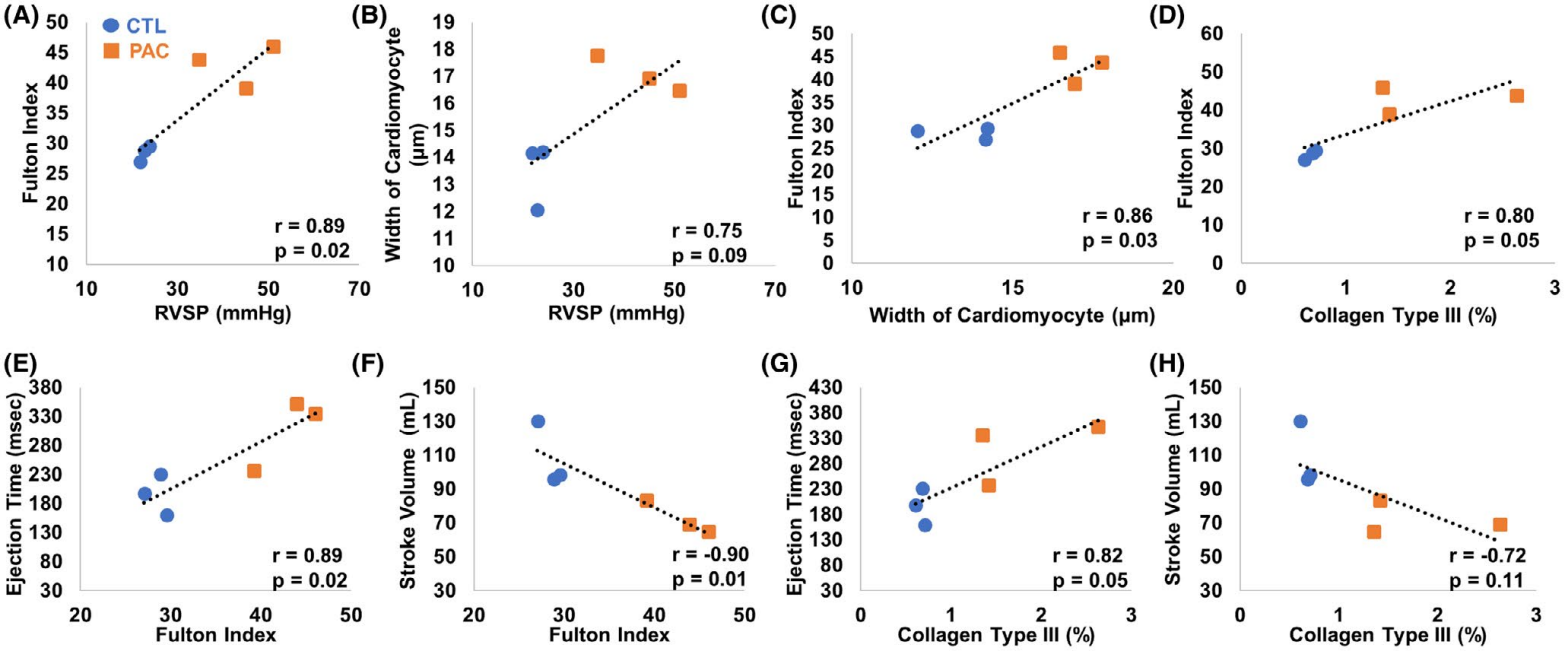
Correlations between the structure and function in the RVs of CTL and PAC groups. A and B, correlations between the RVSP and Fulton index or width of the cardiomyocyte, respectively. C and D, correlations between the width of the cardiomyocyte or the type III collagen content and Fulton index, respectively. E and F, Correlations between the Fulton index and ejection time or stroke volume, respectively. G and H, Correlations between the collagen type III content and ejection time or stroke volume, respectively

We first examined the relations of RV pressure (RVSP) and the structures. As shown in Figure 4A,B, we found a significant correlation between RVSP and Fulton index (*P* < .05), which has been used to indicate RV hypertrophy at the tissue level^23^; there was also a trend of correlation between the RVSP and the width of cardiomyocytes. These correlations indicated that the degree of RV pressure overload was associated with RV hypertrophy at both cellular and tissue levels.

As we found that both Fulton index and width of the cardiomyocyte were correlated with the RVSP, we further investigated the relations between these two hypertrophy indices. It was not surprising to see that there was a significant correlation between the cardiomyocyte width and Fulton index in the RVs of the experimental groups, suggesting that the enlarged cardiomyocytes would contribute to increased RV mass (*P* < .05, Figure 4C). Interestingly, we also found a strong correlation between type III collagen and Fulton index (*P* = .05, *r* = .80, Figure 4D), and this relation was absent between the type I collagen and Fulton index (data not shown). These results indicated that RV fibrosis, especially the accumulation in type III collagen, was correlated with the RV hypertrophy.

Next, we examined the relations between the RV hypertrophy or fibrosis and its function indices. As shown in Figure 4E,F, we found a significant correlation between the Fulton index (hypertrophy) and RV ejection time (ET) and a significant correlation between the Fulton index and the stroke volume, respectively (*P* < .05). Furthermore, we found a significant correlation between type III collagen content and ejection time and a trend of moderate correlation between type III collagen content and stroke volume (Figure 4G,H). However, there were no correlations between type I collagen content and the RV function (data not shown).

## 4 DISCUSSION

In this study, we described a revised PAC ovine model of adult RVF secondary to pressure overload. This model allowed for a customizable constriction between individual animals and at multiple time points. RV hypertrophy and fibrosis were evident in the pressure overloaded sheep. Surprisingly, the increase in type III collagen was more pronounced than the increase in type I collagen. Functionally, RV pressure elevation resulted in declines in RV SV and TAPSE, and progression of chamber dilation and ejection dysfunction, indicating RVF development. The degree of RV hypertrophy and the amount of type III collagen were correlated with the function of the RV.

### 4.1 The revised PAC ovine model of adult RV failure

To date, this study is the first report of an ovine model of adult, chronic RVF. Historically, lambs have been used in the study of RV dysfunction or therapeutics in pediatric patients (Table 1). Since ovine reach sexual maturity at 6‐8 months old,^41^ we explored the potential of RVF establishment in young adults. From the prior literature, it can be found that not all PAC surgeries induced RVF (Table 1). If the PAC induction is too mild, RV adaptation rather than RVF will occur; on the other hand, if the PAC induction is too severe, animal deaths often occur prior to data collection.^42^ In this study, we adopted the same criteria as used previously^30^, ^31^, ^32^, ^34^ to induce a similar degree of hemodynamic insult in these sheep and then examined the remodeling of the RVs. As a result, different (customized) degrees of PAC were induced (Table 2) to ensure that the proper degree of pressure overload was achieved for RVF establishment in different individuals. Therefore, even with a small number of animals (n = 3 per group), we were able to confirm significant structural changes (ie, RV hypertrophy and fibrosis) and functional changes (ie, reduction in systolic function and ejection hemodynamics) in the RVs, from which RVF was evident. This pilot study had 0% of mortality in the PAC animals, which is rare in the similarly reported studies since the model is known for its drawbacks in surgical mortality, especially when the goal is to induce RVF.^42^ Furthermore, while prior preclinical studies examined the development of RVF by either structural or functional changes in the RV, our study examined both aspects comprehensively to fully validate the establishment of RVF in adult sheep.

There are some advantages of the animal‐specific, graded PAC methodology. First, this method allows for a PA constriction that results in identical hemodynamic insult between animals. As shown in Table 1, many preclinical studies used a fixed degree of constriction (ie, increase to certain pressure value, reduce to certain PA diameter, etc) to induce pressure overload. However, each animal responds uniquely to PAC and thus a fixed constriction may lead to varied degrees of RV dysfunction (from adaptation to failure), which may complicate the assessment and diagnoses of RVF among animals. In addition, various degrees of constriction were reported (see Table 1) and there is a lack of guidance on the induction of PAC. Since RV pressure equivalent to systemic pressure has been reported in clinical and preclinical studies of RVF,^43^, ^44^ we decided to use this hemodynamic condition as the criterion of PAC in our model. Indeed, from our own data it can be seen that each animal had its own amount of saline injection to elevate the RV pressure to systemic pressure, suggesting that different thresholds are required to induce RVF in individual animals. The animal‐specific and graded approach also avoids potential unexpected animal death due to a single, severe constriction as we can progressively increase or halt the insult depending on the animal's individual response.

Second, the use of an adjustable PAC method provides more flexibility in the degree of PAC in large animals that is impossible in rodent PAC models. In a rabbit study with a similar PAC method, reversible constriction was induced to investigate the progression and recovery of RVF. This proof of concept study has shown the regression of RV chamber size, hypertrophy, and fibrosis by the removal of pressure elevation, which may support the postulate that RVF is reversible.^45^ Similarly, the graded or reversible PAC could be induced in large animals such as in this ovine model to further investigate the pathogenesis of RVF, including the development from adaptive to maladaptive RV remodeling. Therefore, the model is very flexible and can be adapted to investigate different questions regarding RVF.

### 4.2 New insights of RV failure from the study

In addition to the clearly adverse functional changes, we have observed morphological and structural changes in the RVs in the PAC group that are characteristic of RVF.^10^, ^11^, ^13^ Firstly, RV dilatation and hypertrophy were evident and the RVID/LVID was gradually increased during the progression of RVF (Figure 3C). In a recent study, the ratio of end‐diastolic volumes (EDVs) of the RV to LV (RVEDV/LVEDV) was found to increase with increased RV free wall stiffness in PH patients, and this new index was strongly and inversely correlated with RV peak contractility.^46^ Thus, we speculate that the increased RVID/LVID may indicate a gradual reduction in RV contractility and explain the impaired systolic function (SV) observed in the PAC sheep.

Moreover, RV fibrosis was revealed in the PAC sheep (Table 3). This is not surprising because collagen deposition is universally reported in clinical and preclinical studies, large and small animals, as well as from early to late stage of RVF.^13^, ^23^, ^42^, ^47^ However, it was the collagen type III, not type I, that was more markedly increased in the PAC RVs. This is unexpected because type I collagen is the major isoform of collagen fibers in the RV,^48^, ^49^ and provides more mechanical strength than type III collagen.^50^ We do not know why RVF led to a more significant increase in type III collagen, which will be examined in future investigations. Even in LVs, there is no consensus on whether type I or type III collagen plays a more significant role in its pathogenesis.^37^, ^51^ Future studies should also delineate the role of different subtypes of collagen in RVF.

Finally, we found some interesting correlations among the healthy and failing RVs. RV hypertrophy and fibrosis were strongly correlated with RV function (Figure 4F,H). This indicated that the severity of RV hypertrophy or fibrosis was linearly linked to the adaptation of the RV and could be used as diagnostic parameters indicative of RVF. Indeed, both ventricular mass and collagen deposition have been used in preclinical and clinical settings and were found to correlate with the severity of ventricular dysfunction.^52^, ^53^ These data also confirm that our ovine model recapitulates the behavior and pathogenesis of human RVF. Furthermore, the amount of type III collagen was strongly correlated with the Fulton index (Figure 4D), indicating that certain molecular mechanisms in type III collagen metabolism are linked with RV hypertrophy. To date, the proof of a mechanistic link between fibrosis and RV dysfunction is insufficient.^47^, ^54^ Despite the evidence that increased collagen accumulation is found in severe RVF, the treatment that reversed the collagen deposition in the RV failed to improve the RV function.^54^, ^55^ Here, we observed that type III collagen content was strongly correlated with ejection time and SV (Figure 4G,H). We suspect that the different roles of type I and type III collagen in RV dysfunction may explain the discrepancy in the literature.

### 4.3 Limitations

There are a few limitations to this study. Firstly, we did not have 3D measurements of the RV volume or strain, which are useful indices of RVF.^11^, ^13^, ^56^ Cardiac magnetic resonance imaging or pressure‐volume relations are the gold standard and should be used to investigate adult ovine RVF in the future.^44^, ^57^ Secondly, even though we observed significant functional impairment, other clinical signs such as peripheral edema or body weight loss were absent.^3^, ^13^ These signs are typically seen in the late stage of RVF and the RVF observed in this study may be in an early rather than a late stage.

## 5 CONCLUSION

In this study, we reported a revised animal‐specific, graded pulmonary artery constriction model in adult ovine. The model led to successful right ventricle failure development with significant structural and functional changes as well as some correlations between right ventricle hypertrophy or fibrosis and functional decline. The model is robust and safe to induce various degrees of pressure overload and at multiple time points, which enables the flexibility to adapt to different protocols to answer various research questions related to the progression or treatment of right ventricle failure.

## CONFLICT OF INTEREST

None.

## AUTHOR CONTRIBUTIONS

ZW and EM conceived and designed the study. EM, JB, JE, BN, and MNT performed the experiments and collected the data. MNT and WL performed data analysis and drafted the manuscript. All the authors reviewed, edited, and approved the manuscript.
